# Hemodynamic responses to tracheal intubation with Bonfils compared to C-MAC videolaryngoscope: a randomized trial

**DOI:** 10.1186/s12871-018-0592-7

**Published:** 2018-09-07

**Authors:** Youssef Ezhar, Frederick D’Aragon, Pablo Echave

**Affiliations:** 1 Department of Anesthesiology, Faculty of Medicine and Health Sciences, Universite de Sherbrooke, 3001 12th Avenue N., Sherbrooke, Quebec, J1H 5N4 Canada; 2 Research Centre, Centre Hospitalier Universitaire de Sherbrooke and Faculty of Medicine and Health Sciences, Universite de Sherbrooke, Sherbrooke, Quebec, J1H 5N4 Canada

**Keywords:** Endotracheal intubation, Videolaryngoscope, Bonfils

## Abstract

**Background:**

Direct laryngoscopy (DL) produce tachycardia and hypertension that could be fatal in a patient with a brain injury. Bonfils fiberscope and C-MAC videolaryngoscope are associated with little hemodynamic instability compared to DL. Scientific evidence comparing these two alternatives does not exist. We conducted this study to determine the hemodynamic effects of Bonfils compared to C-MAC in patients undergoing elective surgery.

**Methods:**

Fifty (50) patients listed for elective surgery were randomly assigned to endotracheal intubation with Bonfils or C-MAC. After a standardized induction, intubation was done via the retromolar approach (Bonfils group) or via videolaryngoscopy (C-MAC group). A research assistant, who was not blinded to the intervention, recorded heart rate (HR) and arterial blood pressure (systolic, diastolic and mean arterial blood pressure [MAP]) at induction and at every minute during the 5 min post intubation. The primary outcome was the hemodynamic response to intubation, as verified every minute for the first 5 min compared to baseline value.

**Results:**

After randomization, the two groups were comparable except for ASA I/II ratio which was slightly higher in the C-MAC group (*p* = 0.046). Heart rate (*p* = 0.40) and MAP (*p* = 0.30) were comparable between the two groups within 5 min post intubation. Intubation time was shorter with C-MAC than with Bonfils (30 ± 2 s vs 38 ± 2 s; *p* = 0.02).

**Conclusion:**

Hemodynamic responses to tracheal intubation using the Bonfils fiberscope is comparable to the C-MAC videolaryngoscope among patients scheduled for an elective surgery. In light of these findings, using either technique appears to be a reasonable course of action.

**Trial registration:**

ISRCTN #34923, retrospectively registered, 26/03/2018.

**Electronic supplementary material:**

The online version of this article (10.1186/s12871-018-0592-7) contains supplementary material, which is available to authorized users.

## Background

Direct laryngoscopy can produce marked hemodynamic responses, such as tachycardia and hypertension. This response may be induced by direct contact of the blade with the posterior third of the tongue and the effect of raising the richly innervated epiglottis [1, 2]. More so, as described by Schribman, the insertion of the endotracheal tube between the vocal cords is also responsible for catecholamine release [2]. This hemodynamic response is usually tolerated by healthy patients but can be dangerous, even fatal, in case of brain injury such as subarachnoid hemorrhage [3]. By improving laryngeal view, video laryngoscopes and fiberscopes have proven their utility during novice intubation and difficult airway situations such as cervical spine injuries [4–9]. Can they also be valuable when tight hemodynamic control is deemed necessary? Some practitioners report little hemodynamic modification with Bonfils [10], a rigid fiberscope (Karl Storz, Tuttlingen, Germany) with a 3.5–5.0 mm optical stylet that allows retromolar intubation of the larynx. It is designed to position a 6.5 mm internal diameter (ID) or larger endotracheal tube directly into the trachea with minimal or no manipulation of the epiglottis. Two studies showed reduced hemodynamic effect with Bonfils compared with direct laryngoscopy [10, 11]. C-MAC, a video laryngoscope (Karl Storz, Tuttlingen, Germany) uses a Macintosh blade designed with a camera located at the distal tip of the blade. This study evaluates the effect on heart rate and blood pressure of healthy patients undergoing tracheal intubation using Bonfils or C-MAC.

## Methods

### Design overview

Our hypothesis was that intubation using Bonfils would increase mean arterial pressure and heart rate less than with C-MAC. The study protocol was approved by the Internal Review Board. Between September 2014 and July 2015, 50 patients recruited in our tertiary care centre were randomly assigned to intubation with Bonfils or C-MAC. Written informed consent was obtained from all participants prior to the intervention.

### Study population

Patient selection was based on the following inclusion criteria: elective surgery, American Society of Anesthesia (ASA) 1 or 2, 18–60 years old. Exclusion criteria were: patient refusal, active smoking, chronic hypertension and a history of difficult intubation (Cormack-Lehane grade III or IV), or any of the following criteria: Mallampati > 2, Patil < 4 cm, mouth opening < 3 cm. There was no exclusion criteria related to the medication taken by the patient.

### Intervention

All patients fasted for a minimum of 6 h before the anesthesia and none were allowed to receive fluid repletion before the procedure*.* A peripheral intravenous line (IV) and standard ASA monitoring were installed upon arrival in the operating room. Vital signs were recorded prior to induction and then every minute, up to 5 min after intubation. After pre-oxygenation, induction was initiated using intravenous fentanyl 1–2 mcg/kg, propofol 2–4 mg/kg and rocuronium 0.6–1.2 mg/kg for all patients. After loss of consciousness, bag mask ventilation using 100% oxygen was performed until complete paralysis, as verified by peripheral nerve stimulation of the median nerve. Then, an anesthesiologist or resident proceeded with orotracheal intubation with Bonfils or C-MAC (Storz #8402 ZX) according to randomization. Operators had performed a minimum of 30 intubations with Bonfils and 20 intubations with C-MAC to be eligible. Patients were randomly assigned, through a computer generated sequence, to intubation with Bonfils using retromolar technique or C-MAC in a 1:1 ratio with a permuted block size of 4. The retromolar technique consisted in advancing the endoscope on the right side of the mouth, keeping strictly behind the molar and then tilting toward the operator. Both techniques involved as little airway manipulation as needed to visualize the glottis through the optical lens with the aid of an external monitor. Topical or *IV* lidocaine as well as beta blockers were not used during this protocol. 6.5 ID endotracheal tube (ETT) for women and 7.5 ID for men were used. These ETTs were high-volume / low-pressure, thin-walled, barrel-shaped cuffs made of a polyvinylchloride membrane (PVC). Cuffs were filled with air in order to obtain a seal at a positive ventilation pressure of 30 cmH20. After intubation, sevoflurane was administered in an oxygen/air mixture at 2 l per minute up to 1.0 minimum alveolar concentration target. Sevoflurane was selected over desflurane for its lack of tachycardic effect [12]. Patients were blinded to the group allocation. Staff recording vital signs were not blind to intubation technique.

### Clinical outcomes

Primary outcome was the hemodynamic response to intubation, as verified every minute for the first 5 min post intubation and as compared with initial and 1 min post induction values. Systolic blood pressure (SBP), diastolic blood pressure (DBP) and mean arterial pressure (MAP) were measured using an automatic blood pressure cuff. Heart rate (HR) was followed using a conventional pulse oximeter. MAP variation was defined as the difference between the first minute post induction MAP and the 5 min post intubation MAP. Secondary outcome was the duration of intubation. Duration of intubation was noted and defined as introduction of Bonfils or C-MAC in the oral cavity until confirmation of proper positioning of the endotracheal tube by a positive capnography reading.

### Sample size & statistical analysis

Literature review showed an increase of 20 mmHg in MAP with intubation as clinically significant [3, 10]. Therefore, 32 participants were required to provide a power of 80% for a level of significance of 0.05. Considering potential drop outs and ease of recruitment, 50 patients were recruited. Statistical analysis was done using IBM SPSS Statistics 20 on Windows 7 (Armonk, NY, USA). Chi-square test was used to compare dichotomous variables (e.g. operator, sex, Mallampati score) and Student’s T-test for continuous variables (e.g. weight, height, length of intubation). Mann-Whitney test was used to compare non-parametric variables (e.g. amount of induction medication). A two way repeated measures ANOVA test compared repeat measures, such as HR and blood pressure. *P-*value < 0.05 was considered statistically significant.

## Results

**Fig. 1:**
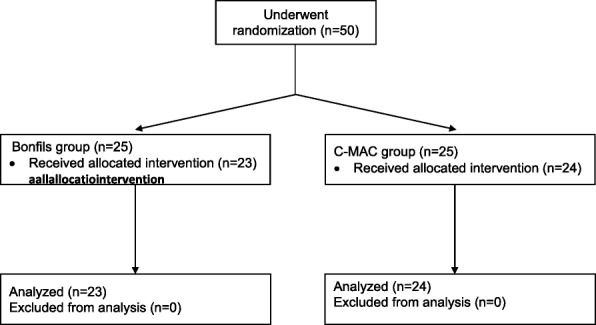
Flow Diagram

**Fig. 2:**
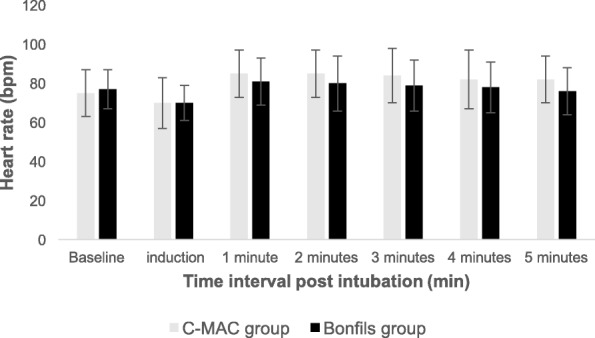
Heart rate during induction and intubation period. Data are expressed as mean ± SD. Bpm: beat per minute, min: minute

**Fig. 3:**
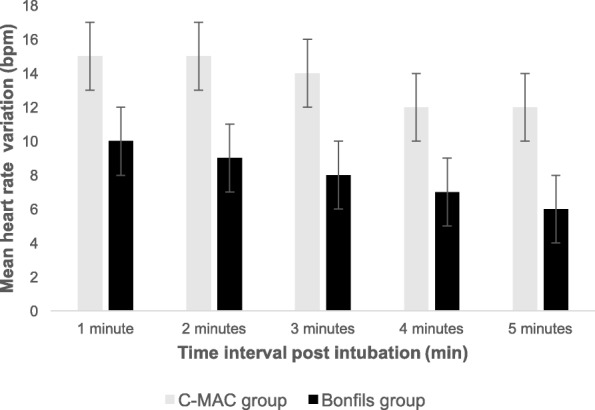
Mean heart rate variation during the post intubation period. Data are expressed as mean ± SD. Bpm: beat per minute, min: minute

**Fig. 4:**
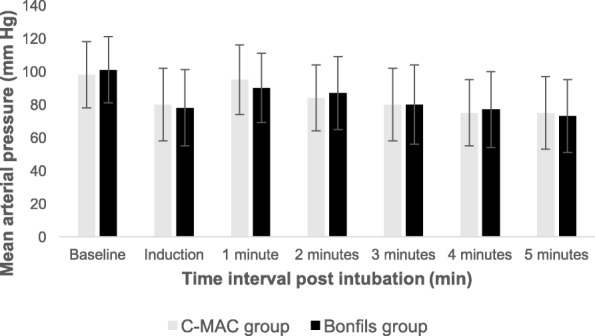
Mean arterial pressure during induction and intubation period. Data are expressed as mean ± SD. Min: minute

**Fig. 5:**
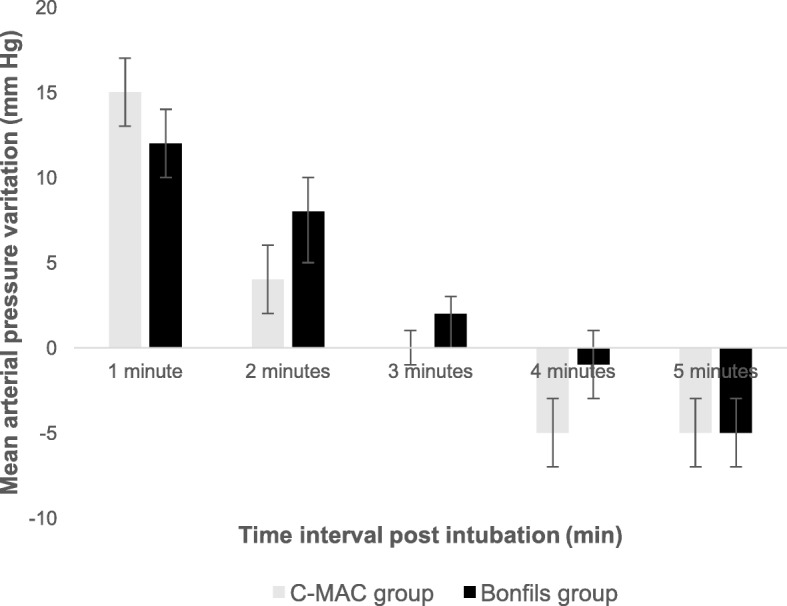
Mean arterial pressure variation during the post intubation period. Data are expressed as mean ± SD. Min: minute

As per Fig. 1, 50 patients were enrolled. No unanticipated difficult intubation occurred. Three patients had protocol violations (2 patients in the Bonfils group and 1 patient in the C-MAC group). They were excluded from the per-protocol analysis but were included for the intention-to-treat analysis Precisely, one patient lost his intravenous access during induction, one because of non-compliance with the drugs induction and one patient was randomized in the C-MAC group but the anesthesiologist used Bonfils for intubation because of the patient’s neck pain. Table 1 compares demographic data, operator’s experience, difficult airway criteria and induction doses. Data were similar in both groups except for ASA I/II ratio which was slightly higher in the C-MAC group. There was no significant difference between groups for HR and MAP in the post intubation period (*p* = 0.40) (Figs. 2, 3 and 4). MAP variation was increased in both groups at one and 2 min post intubation and then declined (Fig. 5). Intubation time (Table 2) was shorter with C-MAC than with Bonfils (30 ± 2 s vs 38 ± 2 s; *p* = 0.02). Results remained similar after including only intubation performed by certified anesthesiologists (Additional file 1). There was no difference between groups for the primary and secondary outcome when analyzed in intention to treat (see Additional file 2). No adverse effects were reported during this study.

**Table 1:** Characteristics of the patients at baseline

Characteristic	C-MAC group (*n* = 25)	Bonfils group (n = 25)	*p* value
Age (yr), mean (SD)	40.6 (10.7)	42.7 (10.6)	NS
Female sex, n (%)	19 (76.0)	22 (88.0)	NS
Weight (kg), mean (SD)	72.3 (13.1)	. 78.7 (16.8)	NS
Height (cm), mean (SD)	165.6 (7.9)	162.9 (5.9)	NS
Body Mass Index	26.4 (4.8)	29.7 (6.4)	NS
Type of surgery n (%):			
Breast surgery	7 (28.0)	8 (32.0)	NS
Hernia repair	3 (12.0)	2 (8.0)	
Gynecologic surgery	10 (40.0)	8 (32.0)	
Cholecystectomy	0 (0.0)	3 (12.0)	
Orthopedic surgery	5 (20.0)	4 (16.0)	
ASA score n (%):			
I	21 (84.0)	11 (44.0)	0.007
II	4 (16.0)	14 (56.0)	
Principal comorbidity n (%):			
COPD	1 (4.0)	1 (4.0)	0.03
Diabetes	0 (0.0)	4 (16.0)	
Obesity (BMI 30–35)	3 (12.0)	9 (36.0)	
Mallampati score n (%):			
I	16 (64.0)	15 (60.0)	NS
II	9 (36.0)	10 (40.0)	
Mouth opening n (%):			
3 cm	16 (62.5)	17 (65.2)	NS
4 cm	8 (33.3)	8 (34.8)	
5 cm	1 (4.2)	0 (0.0)	
Patil score (cm), mean (SD)	6 (1)	6 (0.5)	NS
Fentanyl (mcg), mean (SD)	140.6 (18.5)	146.2 (24.5)	NS
Propofol (mg), mean (SD)	205.0 (39.8)	187.6 (36.8)	NS
Rocuronium (mg), mean (SD)	45.4 (7.5)	45.3 (7.8)	NS
Operator n (%):			
Anesthesiologist	21 (83.3)	15 (60.9)	NS
Resident	4 (16.7)	10 (39.1)

**Table 2:** Intubation time

	C-MAC group (*n* = 24)	Bonfils group (*n* = 23)	*P*-value
Intubation attempt n (%):			
1	20 (83.0)	19 (82.6)	0.62
2	4 (17.0)	3 (13.0)	
3	0 (0.0)	1 (4.4)	
Intubation time (sec), mean (SD)	30.3 (9.3)	38.1 (12.8)	0.02

## Discussion

This study compared the effect of two intubation techniques on hemodynamic parameters. Intubation using either Bonfils or C-MAC induced similar hemodynamic repercussions. However, duration of intubation in the C-MAC group was slightly faster.

Findings in the present clinical trial are statistically and clinically nonsignificant in their differences although there is a lower variation in both hemodynamic parameters in the Bonfils group. Furthermore, neither group exceeded the increase of 20 mmHg in MAP deemed deleterious [3] and HR elevation remained within 10 beats per minute (bpm) of initial value. Our findings could be explained by two reasons. First, evidences suggest less hemodynamic variation with Bonfils. A clinical trial with a similar design randomized 40 patients scheduled for an elective surgery to C-MAC versus Bonfils [13]. Intubation with Bonfils took longer (28.8 +/− 6.6 s vs 24.7 +/− 5.0 s, *p* = 0.02) and was associated with a higher heart rate compared to C-MAC, which is different from our findings. This may be explained by the utilization of a different technique for the insertion of the Bonfils. In our study, we used the retromolar technique. Compared to the midline approach, the retromolar limits the manipulation of the epiglottis and the posterior aspect of the tongue, which are richly innervated in adrenergic receptors [14]. By having a better visualization of the epiglottis we hypothesized that there is a reduction in the sympathetic stimulation leading to tachycardia. Also, as reported previously, the retromolar approach is preferred in patients with limited mouth opening [15]. In addition, we allowed novice operators to performs endotracheal intubation which increase the generalizability of our findings. In this study, C-MAC contacted and elevated the epiglottis, although probably less forcefully than direct laryngoscopy owing to the optic lens, while Bonfils did not. Because both techniques yielded similar hemodynamic variation a natural corollary attributes the increase in HR and MAP mainly to the endotracheal tube going through the trachea, as described by Shribman [2]. Unfortunately, no study compared C-MAC with direct laryngoscopy on hemodynamic parameters [16]. Second, a correlation exists between intubation time and hemodynamic response. Nishikawa et al., demonstrated that a duration of intubation procedure of 40 to 60 s was associated with an increase of 15 to 20 mmHg of the MAP in patients intubated with fiberscope [17]. Taken together, these hypotheses may explain the similar hemodynamic response found with Bonfils and C-MAC.

Hemodynamic instability at induction may be detrimental to patients. It has been demonstrated that an increase of 20 mmHg in MAP is a risk factor for rebleeding in patients with subarachnoid hemorrhage [3]. Even more worrying, an increase in MAP adds a deleterious effect on intracranial pressure, which increases the risk of cerebral herniation. Patients with coronary artery disease are also susceptible to complication secondary to elevated blood pressure or increased heart rate. This was confirmed in one study where healthy patients were compared to patients with stable angina during a stress test. Compromised patients experienced a downward ST segment but not the healthy ones [18].

One Limitation of this study was the small sample size that likely led to an imbalance between groups in baseline variables and limited the interpretation of our results. In addition, we did not control for beta blockers in this study. Although theoretical, obese and diabetic patients are more likely to be on beta blockers and could explain the absence of a higher heart rate in the Bonfils group compared to C-MAC as previously reported [13]. Our population was composed of healthy ASA 1 and 2 patients without known hypertension or tobacco use. Our rationale was that hypertensive patients would have added a confounding physiological variable [10, 12]. Smokers were excluded because they are known to have a high degree of airway responsiveness and pose a significant risk of hypertension with intubation [12]. Difficult airway patients were excluded to ensure a swift intubation technique in all patients and avoid technical bias. This specific population, which may epitomize the classic neurosurgical, trauma, or cardiac patient could be the focus of specific research. Obviously, such a group could also benefit from pharmacological agents such as lidocaine or beta blockers.

Further research is necessary to assess the effect of C-MAC to direct laryngoscopy on hemodynamic parameters. Additionally, studying the effect of the retromolar approach will be useful, since this technique is perceived to be easier to perform.

## Conclusions

In summary, our study demonstrated that the hemodynamic responses to endotracheal intubation with Bonfils fiberscope is comparable to the C-MAC videolaryngoscope in ASA 1 and 2 patients. We observed a significantly shorter time to intubation with the C-MAC approach.

## Additional files

Additional file 1: **Table S3.** Heart rate (bpm) by group with intubation realized by certified anesthesiologists. **Table S4.** Mean arterial pressure by group with intubation realized by certified anesthesiologists (PDF 19 kb)

Additional file 2: **Table S5.** Intubation attempt and mean intubation time in intention to treat analysis. **Table S6.** Heart rate (bpm) by groups in intention to treat analysis. **Table S7.** Mean arterial pressure (mmHg) by group in intention to treat analysis (PDF 27 kb)

## Abbreviations

ASA: American society of anesthesiology
BPM: Beat per minute
DBP: Diastolic blood pressure
DL: Direct laryngoscopy
HR: Heart rate
ID: Internal diameter
IV: Intravenous
MAP: Mean arterial pressure
RCT: Randomized controlled trial
SBP: Systolic blood pressure
