# Supplementary material for: Hemodynamic responses to tracheal intubation with Bonfils compared to C-MAC videolaryngoscope: a randomized trial
Source: BMC Anesthesiol. 2018 Sep 7;18:124. doi: 10.1186/s12871-018-0592-7 (PMC6129002; doi:10.1186/s12871-018-0592-7)
Supplement: Supplementary file 1 — Table S3. Heart rate (bpm) by group with intubation realized by certified anesthesiologists. Table S4. Mean arterial pressure by group with intubation realized by certified anesthesiologists (PDF 19 kb) [file 12871_2018_592_MOESM1_ESM.pdf]

Table 3 : Heart rate (bpm) by group with intubation realized by certified anesthesiologists

Time	C-MAC (n=20)	Bonfils (n=14)
Baseline	74.4 (13.3)	75.2 (12.2)
Induction	68.4 (12.0)	68.7 (12.2)
1 minute	84.0 (15.7)	82.8 (11.2)
2 minute	84.3 (16.8)	79.9 (11.9)
3 minute	83.7 (15.7)	80.0 (11.6)
4 minute	81.6 (14.6)	78.4 (10.9)
5 minute	81.7 (19.2)	75.7 (11.2)

Results are reported as Mean (SD)
p value = 0.559

Table 4 : Mean arterial pressure by group with intubation realized by certified anesthesiologists

Time	C-MAC (n=20)	Bonfils (n=14)
Baseline	99.3 (9.7)	100.1 (12.1)
Induction	99.6 (10.6)	80.9 (11.1)
1 minute	95.6 (19.3)	91.8 (13.6)
2 minute	85.3 (16.3)	84.5 (11.1)
3 minute	80.9 (15.4)	78.1 (8.4)
4 minute	75.3 (13.3)	75.9 (8.8)
5 minute	72.2 (7.9)	74.1 (9.8)

Results are reported as Mean (SD)
p value 0.571
