# Supplementary material for: Hemodynamic responses to tracheal intubation with Bonfils compared to C-MAC videolaryngoscope: a randomized trial
Source: BMC Anesthesiol. 2018 Sep 7;18:124. doi: 10.1186/s12871-018-0592-7 (PMC6129002; doi:10.1186/s12871-018-0592-7)
Supplement: Supplementary file 2 — Table S5. Intubation attempt and mean intubation time in intention to treat analysis. Table S6. Heart rate (bpm) by groups in intention to treat analysis. Table S7. Mean arterial pressure (mmHg) by group in intention to treat analysis (PDF 27 kb) [file 12871_2018_592_MOESM2_ESM.pdf]

Table 5 : Intubation attempt and mean intubation time in Intention to treat analysis

	C-MAC group (n= 25)	Bonfils group (n= 25)	*P*-value
**Intubation attempt n (%):**			
**1**	20 (80.0)	19 (76.0)	0.62
**2**	4 (16.0)	3 (12.0)	
**3**	1 (4.0)	2 (8.0)	
**Mean intubation time (sec)**	38.1 (12.2)	30.3 (9.1)	0.014

Table 6 : Heart rate (bpm) by groups in intention to treat analysis

**Time**	**C-MAC group (n=25)**	**Bonfils group (n=25)**
**Baseline**	75.4 (13.5)	76.4 (12.2)
**Induction**	70.4 (12.7)	70.8 (14.6)
**1 minute**	84.5 (14.8)	80.3 (13.2)
**2 minute**	83.8 (16.4)	79.2 (14.6)
**3 minute**	82.8 (14.7)	77.8 (14.1)
**4 minute**	81.2 (13.8)	76.8 (13.1)
**5 minute**	81.0 (17.7)	75.2 (13.8)

Results are reported as Mean (SD)
p value = 0.364

Table 7 : Mean arterial pressure (mmHg) by group in intention to treat analysis

Time	C-MAC (n=25)	Bonfils (n=25)
**Baseline**	97.2 (11.3)	99.0 (14.6)
**Induction**	79.2 (11.8)	77.8 (10.3)
**1 minute**	93.4 (18.5)	88.4 (14.0)
**2 minute**	83.2 (15.5)	84.9 (15.6)
**3 minute**	79.2 (14.4)	78.6 (12.7)
**4 minute**	74.5 (12.4)	75.5 (11.8)
**5 minute**	74.4 (10.3)	72.2 (11.9)

Results are reported as Mean (SD)
p value = 0.834

Table 6 : Heart rate (bpm) by group with intubation realized by certified anesthesiologists

Time	C-MAC (n=20)	Bonfils (n=14)
Baseline	74.4 (13.3)	75.2 (12.2)
Induction	68.4 (12.0)	68.7 (12.2)
1 minute	84.0 (15.7)	82.8 (11.2)
2 minute	84.3 (16.8)	79.9 (11.9)
3 minute	83.7 (15.7)	80.0 (11.6)
4 minute	81.6 (14.6)	78.4 (10.9)
5 minute	81.7 (19.2)	75.7 (11.2)

Results are reported as Mean (SD)
p value = 0.559

Table 7 : Mean arterial pressure by group with intubation realized by certified anesthesiologists

Time	C-MAC (n=20)	Bonfils (n=14)
Baseline	99.3 (9.7)	100.1 (12.1)
Induction	99.6 (10.6)	80.9 (11.1)
1 minute	95.6 (19.3)	91.8 (13.6)
2 minute	85.3 (16.3)	84.5 (11.1)
3 minute	80.9 (15.4)	78.1 (8.4)
4 minute	75.3 (13.3)	75.9 (8.8)
5 minute	72.2 (7.9)	74.1 (9.8)

Results are reported as Mean (SD)

p value 0.571
